# Training Can Increase Students’ Choices for Written Solution Strategies and Performance in Solving Multi-Digit Division Problems

**DOI:** 10.3389/fpsyg.2018.01644

**Published:** 2018-09-11

**Authors:** Marije F. Fagginger Auer, Marian Hickendorff, Cornelis M. van Putten

**Affiliations:** ^1^Methodology and Statistics, Institute of Psychology, Leiden University, Leiden, Netherlands; ^2^The Netherlands Association of Universities of Applied Sciences, The Hague, Netherlands; ^3^Educational Science, Institute of Education and Child Studies, Leiden University, Leiden, Netherlands

**Keywords:** mathematics, multi-digit arithmetic, division, solution strategies, adaptivity, training

## Abstract

Making adaptive choices between solution strategies is a central element of contemporary mathematics education. However, previous studies signal that students make suboptimal choices between mental and written strategies to solve division problems. In particular, some students of a lower math ability level appear inclined to use mental strategies that lead to lower performance. The current study uses a pretest-training-posttest design to investigate the extent to which these students’ choices for written strategies and performance may be increased. Sixth graders of below-average mathematics level (*n* = 147) participated in one of two training conditions: an explicit-scaffolding training designed to promote writing down calculations or a practice-only training where strategy use was not explicitly targeted. Written strategy choices and performance increased considerably from pretest to posttest for students in both training conditions, but not in different amounts. Exploratory results suggest that students’ strategy choices may also be affected by their attitudes and beliefs and the sociocultural context regarding strategy use.

## Introduction

Tasks are executed using a variety of strategies during all phases of development (Siegler, 1987, 2007; Shrager and Siegler, 1998). For example, infants vary in their use of walking strategies (Snapp-Childs and Corbetta, 2009), first graders in their use of spelling strategies (Rittle-Johnson and Siegler, 1999), and older children in their use of transitive reasoning strategies (Sijtsma and Verweij, 1999). This large variance in strategies goes together with widely differing performance rates of the different strategies, thereby having profound effects on performance levels. As such, strategies have received ample research attention.

Children’s and adults’ strategy use has been investigated for many cognitive tasks, such as mental rotation (Janssen and Geiser, 2010), class inclusion (Siegler and Svetina, 2006), and analogical reasoning (Stevenson et al., 2011). A cognitive domain that has featured prominently in strategy research is arithmetic. Many studies have been conducted on elementary addition (e.g., Geary et al., 2004; Barrouillet and Lépine, 2005), subtraction (e.g., Barrouillet et al., 2008), multiplication (e.g., Van der Ven et al., 2012), and division (e.g., Mulligan and Mitchelmore, 1997; LeFevre and Morris, 1999; Campbell and Xue, 2001; Robinson et al., 2006), which concern operations in the number domain up to 100 that are taught in the lower grades of primary school. Fewer studies have addressed strategy use in more complex multidigit arithmetical tasks in the higher grades, involving larger numbers or decimal numbers (e.g., Selter, 2001; Van Putten et al., 2005; Torbeyns et al., 2009; Schulz and Leuders, 2018). Multi-digit division in particular is an understudied topic. Since many students experience difficulties in this domain, further study into the strategies students use and how these are affected by student and instructional factors is called for (Hickendorff et al., 2010; Robinson, 2017).

### Adaptive Strategy Use

Strategy use in both elementary and multidigit arithmetic consists of different components (Lemaire and Siegler, 1995): individuals’ strategy repertoire (which strategies are used); frequency (how often each strategy is used); efficiency (the accuracy and speed of each strategy); and adaptivity (whether the most suitable strategy for a given problem is used). These four aspects together shape arithmetical performance.

With mathematics education reforms that have taken place in various countries over the past decades (Kilpatrick et al., 2001), *adaptive expertise* has become increasingly important (Baroody, 2003; Hatano, 2003; Verschaffel et al., 2009; McMullen et al., 2016). Adaptive expertise includes flexibility (using various strategies) and adaptivity (selecting the optimal strategy). It contrasts with *routine expertise*, where children apply standard procedures in an inflexible and inadaptive way (Hatano, 2003). Choosing the most suitable strategy for a given problem (i.e., making an adaptive strategy choice) is therefore crucial in contemporary mathematics education.

There are several ways to define adaptivity of a strategy choice, dependent on what is considered the most suitable or “optimal” strategy (Verschaffel et al., 2009). One way is to define adaptivity solely based on task variables: the characteristics of a problem determine which strategy is optimal (e.g., the adaptive strategy choice for a problem like 1089÷11 would be to use the compensation strategy: 1100÷11-1). However, individuals differ in their mastery of different strategies, and the strategy that is most efficient for one person does not have to be the most efficient strategy for another person. A second, more comprehensive, definition of adaptivity therefore also takes individual differences into account: the optimal strategy is the one that is most efficient for a given problem for a particular person. A third definition even includes contextual variables in the definition, such as aspects of the test (e.g., time restrictions and characteristics of preceding problems) and affective aspects of the broader sociocultural context.

Strategy use is not an exclusively cognitive endeavor. Affective factors, like individuals’ beliefs, attitudes, and emotions toward mathematics in general and (adaptive) strategy use in particular, to some extent influenced by the sociocultural context, have been argued to be very important in shaping individuals’ strategy repertoire and choices (Ellis, 1997; Verschaffel et al., 2009). Ellis (1997) identified several affective, sociocultural factors that impact strategy use. Students have an implicit understanding of which ways of problem solving are valued by their community: whether speed or accuracy is more important; whether mental strategies are valued over using external aids; whether using conventional procedures or original approaches is preferred; and whether asking for help in problem solving is desirable.

Given the importance of affective variables (attitudes and beliefs) as determinants of (adaptive) strategy use and the scarcity of research addressing this, further research is called for. We argue that it is theoretically interesting as well as practically highly relevant to investigate in what way the sociocultural context may be manipulated to favorably influence strategy choices. A domain for which this is particularly relevant is multidigit division, since studies reported that students tend to make sub-optimal choices between mental and written strategies for this type of problems (Hickendorff et al., 2009, 2010; Fagginger Auer et al., 2016), which will be elaborated on in the following.

### Strategies for Solving Multi-Digit Division Problems

In mathematics education reform, standard, digit-based written algorithms to solve multi-digit arithmetic problems have a less prominent role than in more traditional mathematics education (Torbeyns and Verschaffel, 2016). In the Netherlands, the traditional algorithm for the operation of division was even abandoned in favor of a new standardized strategy: the whole-number-based approach (Janssen et al., 2005; Buijs, 2008). The major difference between the digit-based algorithm and the whole-number-based approach is whether or not the place value of the digits in the numbers is ignored or respected (Hickendorff et al., 2017; see **Table 1** for examples). That is, in the digit-based algorithm the place value of the digits is ignored (e.g., in **Table 1**, the “54” of 544 is dealt with as 54 and not as 540), whereas the whole-number-based approach respects the place value (e.g., in **Table 1**, 340 is subtracted from 544; Van den Heuvel-Panhuizen et al., 2009). In contemporary mathematics textbooks, the whole-number-based approach is instructed from fifth grade onward, and it is not before sixth grade that the digit-based is instructed (Hickendorff et al., 2017).

**Table 1:** Examples of the digit-based algorithm, whole-number-based approach, and other written strategies applied to the division problem 544÷34.

Digit-based algorithm	Whole-number-based approach	Non-algorithmic strategies
34/544∖16	544:34 =	10 × 34 = 340
34	340 - 10 ×	13 × 34 = 442
204	204	16 × 34 = 544
204	102 - 3×	
0	102	
	102 - 3× +	
	0 16×

Dutch national assessments in 1997 and 2004 showed a decrease in sixth graders’ use of the digit-based algorithm, but use of the whole-number-based approach did not increase accordingly. Instead, students made more use of strategies without any written work (Hickendorff et al., 2009). These mental strategies turned out to be very inaccurate compared to written strategies (digit-based or otherwise), suggesting that suboptimal strategy choices were made. This partly explained the large performance decline that was observed for multidigit division in the assessments (Hickendorff et al., 2009).

In follow-up studies, Fagginger Auer et al. (2016) and Hickendorff et al. (2010) showed that performance improved when writing down calculations was required in (lower mathematical ability) students who spontaneously solved division problems without any written work. This shows that a contextual factor - requiring the use of more efficient strategies - can affect performance favorably in the short term. A valuable next step would be an investigation of instructional contexts that increase students’ *spontaneous choices* for efficient strategies, thereby foregrounding improvements in performance in a more sustainable way than by using test instructions to force students to write down their work.

### Present Study

The present study is intended as a first step of such an investigation. It focuses on (1) the determinants of students’ spontaneous choices between mental and written division strategies and (2) the effect of a training designed to increase students’ choices for written rather than mental strategies, and thereby also their performance. Using a pretest-training-posttest design, an explicit-scaffolding training condition designed to promote writing down calculations was compared to a practice-only training condition where strategy use was not explicitly targeted. The explicit-scaffolding training involved a step-by-step problem-solving plan for multi-digit division problems, based on the principles of direct, explicit instruction that lower-ability students tend to profit from (Kroesbergen and Van Luit, 2003; Gersten et al., 2009). The practice-only training involved practicing problem solving only, without explicit scaffolding, but with feedback on the accuracy of the outcome as in the explicit-scaffolding condition.

The study focuses on sixth graders of below-average mathematics achievement level. We focused on sixth graders since in the Netherlands instruction in standardized written strategies begins in grade five. Therefore, sixth graders are likely to have experience with written strategies which would be a prerequisite to choose them. After grade six students enter secondary school, where other aspects of mathematics are central to instruction and practice. We focused on below-average achievers because these students tend to be more inclined to use mental strategies than their higher-achieving peers, whereas they have the lowest performance with mental strategies (Hickendorff et al., 2010; Fagginger Auer et al., 2016). In other words: with these students there is most need for, as well as most room for, improvement.

The study aimed to address three sets of research questions and accompanying hypotheses. Research question 1 was: to what extent are individual differences in strategy choice (mental vs. written) related to students’ attitudes and beliefs toward mathematics in general and toward strategies in particular, and to aspects of the sociocultural context of the students’ mathematics classroom (mathematics instruction, teacher attitudes and beliefs)? This investigation is exploratory in nature, and therefore we did not formulate *a priori* hypotheses.

Research question 2 was: to what extent do the two training types affect students’ strategy choice? Hypothesis 2a was that written strategy choices increase more from pretest to posttest in the explicit-scaffolding training than in the practice-only training. Hypothesis 2b was that the effects of the explicit-scaffolding training on the use of written strategies is larger for boys than girls, since boys tend to use more mental strategies in division than girls (Hickendorff et al., 2009, 2010; Fagginger Auer et al., 2013).

Research question 3 was: to what extent do the two training types affect students’ performance? Hypothesis 3a was that performance increases from pretest to posttest in both training types since students in both conditions can practice solving division problems and receive outcome feedback. Hypothesis 3b was that the performance increase in the explicit-scaffolding training is larger than in the practice-only training, as a corollary of the expected increase of written strategies in the former group. Furthermore, within the explicit-scaffolding training, we expect to find different performance gains with regard to students’ gender, mathematical ability level and working memory capacity (hypothesis 3c–3e). Hypothesis 3c was that the performance gains are larger in boys than in girls, as a corollary of the expectation that boys show a larger increase in written strategies use (cf. hypothesis 2b). Hypothesis 3d was that performance gains are larger for students with lower compared to higher mathematical ability level, because mental strategies are especially inaccurate for lower ability students (Hickendorff et al., 2010; Fagginger Auer et al., 2016. Finally, Hypothesis 3e was that training has a larger effect on performance when students’ working memory capacity is lower, since mental strategies demand working-memory resources. Freeing up those resources by writing down calculations may therefore have a larger impact in students with lower working-memory capacity (in line with cognitive load theory; Paas et al., 2003). This is especially relevant in our sample, given that students with a lower mathematical ability tend to have a lower working memory capacity than higher ability students (Friso-van den Bos et al., 2013).

## Materials and Methods

### Participants

In total, 19 different classes of 15 different schools agreed to participate. The schools were located in different medium-sized to large cities in the megalopolis in central-west Netherlands (the Randstad) and from one smaller city in east Netherlands.

There were 323 sixth graders in total, of whom 186 students had a percentile score below 50 on the most recent standardized national mathematics test (Janssen et al., 2010). Furthermore, students with a percentile score below 10 (*n* = 39) were excluded because atypical problems such as dyscalculia could occur in this group. Our effective sample of below-average achievers (percentile score between 10 and 50) thus contained 147 students (64 percent girls; mean age 11 year 9 month with *SD* = 5 month). These students were assigned to one of the two training conditions using random assignment with gender, ability quartile and school as blocking variables: 74 received explicit-scaffolding training and 73 practice-only training.

The 19 teachers of the students (8 female) were on average 38 years old. Four different textbooks were used across the classes: Wereld in Getallen (9 classes), Pluspunt (5 classes), Alles Telt (4 classes), and Rekenrijk (1 class).

### Materials

#### Pretest and Posttest

The pretest to assess students’ division strategy choices and performance contained twelve multidigit division problems presented in **Table 2**. These problems were selected from the two most recent national assessments of mathematical ability at the end of primary school (Janssen et al., 2005; Scheltens et al., 2013), so that they resemble the type of problems students are used to solving (ecological validity). All problems were situated in realistic problem solving context (e.g., determining how many bundles of 40 tulips can be made from 2500 tulips), except for the problem 31.2÷1.2. The test also contained twelve problems involving other mathematical operations (all from the most recent national assessment) as filler items. The posttest was identical to the pretest to allow for a direct comparison of results. Since the pretest and posttest were a month apart and students are used to solve arithmetic problems on a daily basis in their mathematics lessons during that period, it was very unlikely that students remembered any of the (rather complex) solutions.

**Table 2:** The division problems in pretest and posttest. Problems presented in italics are parallel versions of the problems that are not yet released for publication.

Number	Problem
1.	1536÷16 = 96
2.	872÷4 = 218
3.	*31.2*÷*1.2* = *26*
4.	*6496*÷*14* = *464*
5.	*544*÷*34* = *16*
6.	11585÷14 = 827.5
7.	*47.25*÷*7* = *6.75*
8.	157.50÷7.50 = 21
9.	2500÷40 = 62
10.	1470÷12 = 122.50
11.	736÷32 = 23
12.	16300÷420 = 39

Prior to the pretest and the posttest students received an instruction in which the experimenter explained that the students had to do a booklet with mathematics problems. The researcher explicitly stated that this was not a test but that (s)he was interested in learning more about how students go about solving such problems. Furthermore, students were instructed that if they wanted to write down calculations, they could do so in the booklet.

After students completed the mathematics problems in the booklets, the accuracy of the answer (correct or incorrect) and use of written work (yes or no) were scored for each problem. Solutions with written work were further classified into one of three strategy categories: the digit-based algorithm, the whole-number-based approach, and other written strategies (see **Table 1** for examples).

#### Training Problems

The problems used in the three training sessions between the pretest and posttest were three sets of parallel versions of the twelve problems in **Table 2**.

#### Student and Teacher Questionnaires

The students filled out a questionnaire of seven questions (**Appendix A**) on their attitudes and beliefs toward mathematics in general and strategies in particular. The teachers filled out a questionnaire of fifteen questions (**Appendix B)** on their instructional practices regarding standardized division strategies, and attitudes/beliefs toward the importance of writing down calculations and various aspects of flexible and adaptive strategy use. The student and teacher questionnaires were devised specifically for this study.

#### Working Memory Tests

Students’ working memory capacity was assessed using a computerized version of the digit span test from the WISC-III (Stevenson and De Bot, unpublished; Wechsler, 1991), and their spatial working memory using a computerized version of the Corsi block test (Corsi, 1972).

### Training

In the training sessions, students worked on the set of training problems for that week. The experimenter evaluated each answer when it was written down and told the student whether it was correct or incorrect. When correct, the students proceeded to the next problem. When incorrect, the student tried again. Accuracy feedback was provided again, and regardless of whether the solution was correct this time, the student proceeded to the next problem. The session was terminated when 15 min had passed.

Two aspects differed between the two training types. First, students in the practice-only training were free in how they solved the problems (just as in the pretest), whereas the students in the explicit-scaffolding condition had to write down their calculations in a way that “would allow another child to see how they had solved the problem” (but apart from that, the choice for which type of written strategies was free). Second, when students in the practice-only condition failed to provide the correct answer in their first problem-solving attempt, they did not receive any feedback other than that the answer was incorrect before they could try to solve the problem in the second attempt. By contrast, when students in the explicit-scaffolding condition failed to provide the correct answer the first time, they were provided with explicit systematic scaffolding how to write down their calculations in a standardized way at the second attempt. A printed version of this step-by-step plan was always on the table so that students could use it whenever they wanted. When students were stuck in their problem solving, the experimenter used the plan and standardized verbal instructions to help the students with writing down calculations. No feedback was given on the accuracy of what students wrote down (e.g., mistakes in the multiplication table), except for the final answer.

**FIGURE 1:**
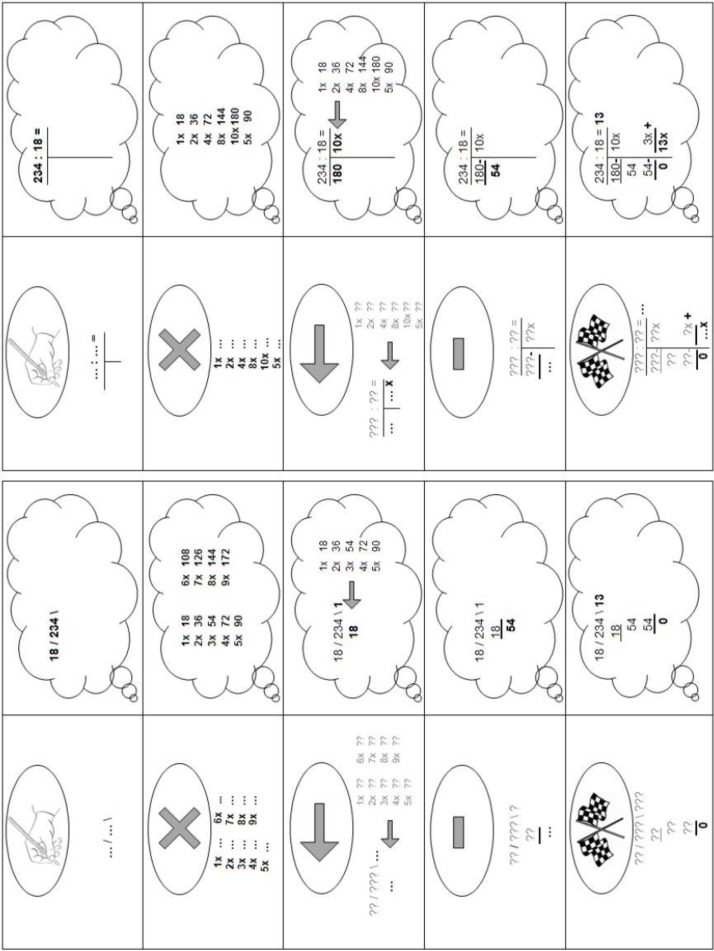
The step-by-step plans in the explicit-scaffolding training in two versions: for students using the digit-based algorithm, and for students using the whole-number-based approach.

Since classes differed in which type of standardized strategy was instructed, there were two versions of the plan: one for students taught the digit-based algorithm and one for students taught the whole-number-based approach (see **Figure 1**). In cases where students were taught both standardized strategies, the experimenter showed both step-by-step plans and the student could select the strategy (s)he was used to applying. Both versions consist of five highly similar steps (with step 3 and 4 repeated as often as necessary): (1) writing down the problem; (2) writing down a multiplication table (optional step); (3) writing down a number (possibly from that table) to subtract; (4) writing down the subtraction of that number; and (5) finishing when zero is reached, which in the case of the whole-number-based approach requires a final addition of the repeated subtractions. Each step was represented by a symbol to make the step easy to identify and remember (the symbols in the ellipses on the left side of the scheme). Below this symbol, a general representation of the step was given, with question marks for problem-specific numbers already present at that step and dots for the numbers to be written down in that step. On the right-hand side of the plan, an example of the execution of each step for the particular problem 234÷18 was given in a thinking cloud. On both sides, the elements to be written down in the current step were in bold font.

### Procedure

The study was conducted over a period of 5 weeks in the fall. In week 1, students first completed the pretest in their classroom, in a maximum of 45 min, and also the two working memory tasks (on the computer) and the student questionnaire. In week 2–4, students participated in three individual training sessions of 15 min each (one per week) with an experimenter. The experiment was concluded in week 5, in which students completed the posttest. The teacher filled out the teacher questionnaire in week 1.

### Statistical Analysis

#### Research Question 1

Correlations were used to explore relations between students’ percentage of written strategy choices across the twelve pretest problems on the one hand and (a) student factors (attitudes and beliefs, based on student questionnaire) and (b) classroom factors (mathematics educational practices and sociocultural context, based on teacher questionnaire) on the other. These were point-biserial correlations for dichotomous questionnaire responses and Spearman’s rank correlations for scales.

### Research Questions 2 and 3

Explanatory IRT models (De Boeck and Wilson, 2004) were used to model the effect of the training types on pretest-posttest differences in strategy choice (question 2) and in performance (question 3), as well to investigate differential training effects by students’ gender, mathematical ability level, and working memory. Measuring learning and change has inherent problems (Embretson and Reise, 2000; Stevenson et al., 2013). For instance, the interpretation of change scores depends on the score at pretest (e.g., a change from 1 to 3 may not mean the same as a change from 6 to 8), because sum scores in general and change scores in particular are not of interval measurement level. IRT models place persons and items on a common latent scale, resulting in a higher likelihood that the persons’ ability estimates are of interval measurement level than simple sum scores (Embretson and Reise, 2000). To answer research question 2, the dependent variable of the IRT models was strategy choice (written vs. not written) on each problem of the pretest and posttest, whereas it was accuracy of the answer (correct vs. incorrect) in the analyses to answer research question 3.

IRT models can be extended with an explanatory part by including explanatory variables, which can be item factors, person factors, and person-by-item factors. The current analyses included the following person factors: students’ training condition, gender, mathematical ability score, and working memory. The person-by-item factor solution strategy choice (mental vs. written) was included in research question 3 only.

(Explanatory) IRT models can be estimated as multilevel logistic regression models, using general purpose software for generalized linear mixed models (GLMM) (De Boeck and Wilson, 2004). In the present study, the models were fitted using the lme4 package in R (De Boeck et al., 2011; Bates et al., 2014). All models were random person-random item Rasch models (RPRI; De Boeck, 2008), with a random intercept for students, and also a random intercept for items (as the problems were considered a draw from the larger domain of multidigit division). The explanatory variables were added in stepwise fashion (as in Stevenson et al., 2013, see also Pavias et al., 2016), allowing evaluation of the added value of each step by comparing the models based on the Akaike Information Criterion (AIC), Bayesian Information Criterion (BIC), and likelihood ratio tests. The AIC and BIC balance model fit and parsimony (lower values are better). The likelihood ratio test (LRT) statistically tests the added value of including a specific explanatory variable by testing whether the more complex model with this specific explanatory variable included fits significantly better than the less complex model (without that variable).

For an indication of the size of significant effects, the probability of using a written strategy (question 2), or providing a correct answer (question 3), is computed for different levels of the explanatory variable of interest (with all other explanatory variables in the model set at the sample mean in the sample). For example, for the effect of testing occasion (pretest or posttest), the probability of a correct answer for an average student on an average problem on both the pretest and the posttest is computed. For scale variables (e.g., mathematics ability score) the effects of a difference of one standard deviation around the mean (*M*-0.5*SD* to *M*+0.5*SD*) are given.

## Results

### Research Question 1: Determinants of Written Strategy Choices

Students used written strategies in 59 percent of their pretest solutions, which varied across problems between 33 percent (31.2÷1.2) and 76 percent (544÷34), and across students between 0% (*n* = 13) and 100% (*n* = 15). In the following we report correlations between students’ percentage of written strategy choices across the twelve pretest problems on the one hand, and (a) student factors (attitudes and beliefs) and (b) classroom factors (mathematics educational practices and sociocultural context) on the other.

#### Student Factors

**Appendix A** shows the frequencies of the students’ (*n* = 147) responses to the questionnaire items regarding their mathematical attitudes and beliefs, and the correlation of these responses with their percentage of written strategy choices at pretest. In the following, only significant correlations are discussed.

On average, the students had a slightly positive attitude toward mathematics (*M* = 2.8 on a 5-point scale), reported putting quite some effort into math (*M* = 4.3), were slightly positive about their mathematical ability (*M* = 2.8), and almost all students (97 percent) reported valuing accuracy over speed. These factors were not significantly related to written strategy choices. On the questions concerning strategy use, a majority of students (77 percent) found it more important to be able to solve mathematical problems with rather than without paper, and this was positively related to using written strategies (*r* = 0.23). Students reported that they sometimes answer without writing down a calculation (*M* = 2.6) and this self-reported frequency of non-written strategies was negatively related to using written strategies at pretest (*r* = -0.19). When asked to select their reasons to not write down calculations (multiple answers possible), the most frequently reported reason (selected by 49 percent of students) was because they “did not feel it was necessary,” followed by because “it was faster” (41 percent). Other reasons were because of “not feeling like it” (22 percent), because they “guessed the solution rather than computing it” (14 percent), because “mental strategies are more accurate” (14 percent), and because “it is smarter to be able to solve a problem mentally” (11 percent). Virtually no students (1 percent) reported they used a mental strategy because “it was cooler.”

#### Classroom Factors

**Appendix B** shows what the 19 teachers reported on the teacher questionnaire. With the exception of one item, the teachers’ responses were unrelated to their students’ use of written strategies. Most teachers taught their students only the whole-number-based approach exclusively (*n* = 11) or in combination with the digit-based algorithm (*n* = 5); three teachers taught their students the digit-based algorithm exclusively. On average, teachers did not prefer one standardized strategy over the other (*M* = 3.0), but did prefer the use of standardized over non-standardized approaches (*M* = 2.2). Only this item correlated with students’ use of written strategies: the more teachers preferred non-standardized strategies, the lower the percentage of their students’ written strategies (*r* = -0.46). On average, teachers found performing calculations well on paper and mentally equally important for their students (*M* = 3.0). They reported instructing their students in writing down calculations frequently (on average almost daily, *M* = 4.2). Concerning multidigit division problems specifically, teachers on average found writing down calculations somewhat more important for their students than trying to do it mentally (*M* = 2.4) and valued accuracy somewhat over speed (*M* = 2.5). Making a good estimation of the solution was valued more than being able to determine the exact solution (*M* = 3.5), as was knowing more solution procedures rather than just one (*M* = 3.4). Teachers considered using a standardized approach versus choosing a custom solution strategy on average equally important (*M* = 3.0), and valued convenient shortcut strategies somewhat more than using an approach that can always be applied (*M* = 3.3).

### Research Questions 2 and 3

#### Descriptive Statistics

**Table 3** presents descriptive statistics about the content of the training. As instructed, students in the explicit-scaffolding condition virtually always wrote down a calculation (98–99 percent). Though not instructed to do so, students in the practice-only condition also had a high and increasing tendency to use written strategies (81–93 percent). The feedback in the explicit-scaffolding condition (on average 3.3 times per session) included writing down a multiplication table (0.8 times), selecting a number from that table (1.1 times), writing down of the problem (0.5 times), subtracting the selected number (0.5 times), and finishing the procedure (0.5 times).

**Table 3:** Descriptive statistics of training sessions (averages across students).

Training	Number of problems per session	Number of second attempts per session	Feedback Frequeny per session	% Written strategies		
				Session 1	Session 2	Session 3
Explicit scaffolding	5.1	1.6	3.3	98	99	99
Practice-only	6.1	1.8	–	81	87	93

#### Research Question 2

The effects of the training on written strategy choices were evaluated using a series of explanatory IRT models on the pretest and posttest data, with successively more explanatory variables (see **Table 4**). First a baseline model for the probability of a written strategy choice was fitted with only random intercepts for students and problems and no covariates (model *M*_0_). In model *M*_1_, main effects were added for the student characteristics gender, mathematical ability and working memory capacity, which improved fit according to all criteria. Fit was further improved by adding a main effect for testing occasion (pretest vs. posttest; model *M*_2_). However, the change in written strategy choices from pretest to posttest did not significantly differ for the two training groups (model *M*_3_). Adding interactions between condition, testing occasion and student characteristics also did not improve the model (models are not included in **Table 4**).

**Table 4:** Explanatory IRT models for effects on written strategy choices (all comparisons are to *M*_n-1_).

Model	Added fixed effects	LL	# Parameters	AIC	BIC	Likelihood Ratio Test
*M*_0_		-1337.6	3	2681.1	2699.4	
*M*_1_	Gender, math ability, and working memory	-1315.7	6	2643.3	2679.8	χ^2^(3) = 43.8, *p* < 0.001
*M*_2_	Testing occasion	-1216.5	7	2447.0	2489.5	χ^2^(1) = 198.3, *p* < 0.001
*M*_3_	Condition × occasion	-1215.6	9	2449.2	2503.9	χ^2^(2) = 1.7, *p* = 0.42

Interpretation of the best fitting model, *M*_2_, shows that girls used more written strategies (*P* = 0.94) than boys (*P* = 0.74), *z* = -6.0, *p* < 0.001, and that mathematical ability score was positively associated with using written strategies (*P* = 0.80 vs. *P* = 0.92 for one standard deviation difference), *z* = 4.3, *p* < 0.001. Working memory (sum score of the verbal and spatial working memory scores) had no significant effect, *z* = -0.6, *p* = 0.55. Students used more written strategies at the posttest (*P* = 0.94) than at the pretest (*P* = 0.76), *z* = 13.5, *p* < 0.001.

To investigate whether the two trainings differ in the type of written strategies they elicited, **Table 5** presents a more detailed categorization of strategies than just written or non-written. It shows that the frequency of using the digit-based algorithm and whole-number-based approach, other written strategies, non-written strategies and other strategies is almost identical (differences of no more than five percentage points) in the two training groups - both at pretest and at posttest. In both groups, similar increases in the use of both types of standardized strategies and decreases in the use of other written and non-written strategies occurred.

**Table 5:** Strategy use proportions on the pretest and posttest in the different training conditions.

	Pretest		Posttest	
	Explicit-scaffolding	Practice-only	Explicit-scaffolding	Practice-only
Digit-based algorithm	0.09	0.09	0.13	0.13
Whole-number approach	0.37	0.40	0.61	0.62
Other written	0.19	0.19	0.13	0.08
No written work	0.35	0.30	0.13	0.17
Remainder	0.01	0.02	0.00	0.00

#### Research Question 3

Model fit statistics for performance (accuracy) are presented in **Table 6**. As for written strategy choices, first a baseline model for the probability of a correct response was fitted (*M*_0_), and again, this model was improved by adding student gender, ability and working memory (*M*_1_) and by adding testing occasion (*M*_2_), but not by adding condition effects (*M*_3_). The best fitting model, *M*_2_, shows that girls (*P* = 0.43) performed better than boys (*P* = 0.28), *z* = -3.8, *p* < 0.001, and that general mathematics ability score was positively associated with performance (*P* = 0.28 vs. *P* = 0.43 for one SD difference), *z* = 4.5, *p* < 0.001. Working memory had no significant effect, *z* = 0.04, *p* = 0.97. Students performed better at the posttest (*P* = 0.48) than at the pretest (*P* = 0.24), *z* = 11.9, *p* < 0.001.

**Table 6:** Explanatory IRT models for effects on accuracy (all comparisons are to *M*_n-1_).

Model	Added fixed effects	LL	# Parameters	AIC	BIC	Likelihood Ratio Test
*M*_0_		-1801.0	3	3607.9	3626.1	
*M*_1_	Gender, math ability, and working memory	-1785.3	6	3582.5	3619.0	χ^2^(3) = 31.4, *p* < 0.001
*M*_2_	Testing occasion	-1711.1	7	3436.3	3478.8	χ^2^(1) = 148.3, *p* < 0.001
*M*_3_	Condition × occasion	-1710.8	9	3439.6	3494.2	χ^2^(2) = 0.7, *p* = 0.70

Next, the difference in accuracy between written and non-written strategies was investigated by fitting a model for accuracy with main effects for all previous predictors (student characteristics, testing occasion, and condition) and strategy choice (written or not), and all first-order interactions between strategy choice and the other predictors. This showed that written strategies were much more accurate (*P* = 0.40) than non-written strategies (*P* = 0.19), *z* = 4.1, *p* < 0.001, and that this did not depend significantly on testing occasion, *z* = 1.1, *p* = 0.27, gender, *z* = 0.0, *p* = 0.99, ability, *z* = 1.0, *p* = 0.32, working memory, *z* = 0.3, *p* = 0.75, or condition, *z* = -1.0, *p* = 0.33. Finally, we investigated the extent to which individual students’ gains in written strategy choices from pretest to posttest were related to their gains in accuracy from pretest to posttest. Spearman’s rank correlation between difference in written strategy use and difference in accuracy was significant and positive: *r*(142) = 0.23, *p* = 0.006. These results show that not only written strategies are more accurate than mental ones, but also that increasing the use of written strategies leads to increased performance.

## Discussion

The current study’s aim was to investigate determinants of below-average sixth graders’ choices between mental and written strategies for solving multi-digit division problems, and the effect of a training to increase students’ choices for written rather than mental strategies. First, exploratory analyses showed that individual differences in strategy choice (mental vs. written) were related to some aspects of students’ attitudes and beliefs toward strategy use, but not to their attitudes and beliefs toward mathematics in general. Specifically, students who reported that it is more important to solve problems with rather than without paper, and students who reported not so often using non-written strategies were more inclined to use written strategies at the pretest items. Students’ individual differences in strategy choice were related to only one aspect of the sociocultural context (as measured with a teacher questionnaire): the more teachers valued standardized over non-standardized strategies, the more their students used written strategies. An important remark is that since there were only 19 teachers in our sample, low statistical power may have prevented finding other significant associations. Furthermore, the students were instructed by their current teacher for only 2–4 months, which could be another explanation that there were hardly any relations found between teachers’ instructional practices and students’ strategy use. Overall, teachers reported frequent instruction in writing down calculations, preferred use of a standardized over a non-standardized strategy, and valued written strategies somewhat over mental strategies and accuracy somewhat over speed. These results suggest a sociocultural context in which there is room for written strategies, but where it is not the highest priority.

In the second part of the study, the effects of a training designed to promote students’ choices for written rather than mental strategies (and thereby, their performance) were compared to the effects of a practice-only training. In both training conditions the use of written strategies and accuracy increased from pretest to posttest, written strategies were more accurate than mental ones, and individual students’ increase in the use of written strategies was related to their performance gains. However, the hypothesized differential training effects were not observed. Students’ written strategy choices increased to the same extent in both training conditions (in contrast with hypothesis 2a) and there were no differential training effects for boys and girls (in contrast to hypothesis 2b). Regarding performance, performance (accuracy) increased in both groups from pretest to posttest (in line with hypothesis 3a), but not more so in the explicit-scaffolding training condition (in contrast to hypothesis 3b). Furthermore, there were no differential performance gains by gender, mathematical ability level, or working memory (in contrast to hypotheses 3c–3e).

All in all, written strategy choices and performance were considerably higher after training than before training, irrespective of the type of training. Both training types were thus effective in increasing the use of written strategies and thereby performance. However, the elements of explicit scaffolding written strategy use did not add to the effect of only practicing solving the problems with outcome feedback. While writing down calculations was not required during practice-only training, it did occur frequently and increasingly across the training sessions. In the first session calculations were written down in 81 percent of the problems - considerably more than the 70 percent during the pretest. This increased up to 93 percent in the third training session, whereas it decreased to 87 percent in the posttest again. As such, students practiced written calculations almost as much in the practice-only training as in the explicit-scaffolding training, reducing the contrast between the two conditions. The common elements of both trainings – practicing written strategies with outcome feedback – therefore seem to account for the observed changes in strategy choices and accuracy.

In the practice-only condition, the relatively high frequency of written strategy choices in the training sessions compared to the pretest and posttest may possibly be explained by differences in the setting: in a classroom (at pretest and posttest) versus one-on-one with an experimenter (training sessions). Previous research showed a similar difference between a classroom administration setting and individual testing (Van Putten and Hickendorff, 2009). A possible explanation is that students use written strategies because they think the experimenter may expect or prefer that (i.e., demand characteristics; Orne, 1962), in line with the students’ teachers’ light preference of written over mental strategies.

The increase in the use of written strategies over the three training sessions in the practice-only training may possibly be explained by the direct accuracy feedback after each solution (Ellis et al., 1993), and the requirement to do a problem again when the first solution was incorrect. Direct accuracy feedback allows for an immediate evaluation of the success of the strategy that was applied, and this evaluation should often be in favor of written rather than mental strategies given the considerably higher accuracy of the former. Combined with the extra effort associated with an incorrect solution (redoing the problem), this is likely to be an important incentive for written strategy choices.

The element that was unique to the explicit-scaffolding training was the requirement to use a written strategy, scaffolded by a step-by-step plan for writing down calculations. The finding that this element apparently did not have an additional effect contrasts with the results of a meta-analysis on mathematics interventions for low-ability students that identified such plans as an important component of effective interventions (Gersten et al., 2009). In the current study students turned out to require little feedback based on the plan, and the feedback that was given most often concerned an optional element: the multiplication table. Furthermore, students in the practice-only training turned out to practice solving on average one problem more compared to students in the explicit-scaffolding training, which may have masked potential positive effects of the scaffolding elements (similar to Van de Pol et al., 2015).

In addition to the finding that there were no differences in the effects of the two training types, also no differential training effects by gender, mathematical ability and working memory were found. This may be explained by the same reasoning: in practice the difference between the two training types may have been much smaller than intended.

### Limitations

There are several limitations that deserve attention. First, there was no genuine control group of students who did not receive training. Therefore it is not possible to ascribe with certainty the gains in written strategy use and performance to the training. We did, however, collect pretest and posttest data from the 137 students with above-average mathematics achievement level who were in the participating classes, but did not participate in any of the trainings. The pretest-to-posttest increase in both the use of written strategies and in performance was significantly higher in the (below-average achieving) students who received training than in the (above-average) students who did not receive training This differential learning effect supports confidence in the interpretation that it was the training that was effective in increasing written strategy use and performance, although the difference in achievement level between the two groups (below-average vs. above-average) possibly confounds this effect.

A second limitation is that there was no retention test. It was therefore not possible to analyze the stability of the trainings’ effects. Future studies should include a follow-up test later in the school year to address this specifically.

A third limitation concerns the measurement of the teacher’s instructional practices. The use of a questionnaire may not present a complete picture of the actual instructional activities taking place in the mathematics classroom (Porter, 2002), and future studies should include classroom observations to measure the instruction in a more direct way. Moreover, the amount of time the students were instructed by their teacher was relatively short (2–4 months) possibly weakening the effect the teacher’s instructional practices may have had. Future studies could be conducted in the second half of the school year so that the students have received instruction from their teacher for a longer period of time.

### Future Directions

The results of the present study provide several suggestions for future research on strategy training programs. The results suggest that direct accuracy feedback (possibly with some cost attached to incorrect solutions) may be conducive to beneficial changes in strategy choices. They also show that considerable changes in strategy choices and improvements in performance may be achieved with as few as three training sessions of 15 min (in line with the finding of Kroesbergen and Van Luit, 2003, who found that longer mathematics interventions are not necessarily more effective). As said, a follow-up test after a longer period of time (e.g., several months) should be used to establish whether the changes are lasting.

The results also provide suggestions for other possible ways to influence students’ choices between mental and written strategies. Since strategy choices appear to be related to students’ valuing of written strategies and to teachers’ valuing of standardized over non-standardized strategies, a sociocultural context that highlights these aspects may affect strategy students’ strategy choice (Ellis, 1997). This might be achieved by having teachers express more appreciation of the use of external aids in problem solving and of standardizing written solution steps.

## Conclusion

The present study showed that three training sessions in which students practice solving division problems with written strategies and receive feedback on the accuracy of the outcome, whether or not explicitly scaffolded with a step-by-step direct-instruction plan, increased below-average sixth graders’ use of written strategies and performance in solving multi-digit division problems. Given the fact that students seem to make sub-optimal choices for non-written strategies in this domain, this is an important starting point for efforts to increase the use of written strategies. Further research is necessary to identify the optimal set-up of a training targeting students’ written strategy use.

## Ethics Statement

This study was carried out in accordance with the recommendations of ethical guidelines of the Ethics Committee of the Institute of Psychology, Leiden University. The protocol was approved by the Leiden University Psychology Research Ethics Committee (CEP number 6520034071). All subjects gave written informed consent in accordance with the Declaration of Helsinki.

## Author Contributions

MFA, MH, and CvP contributed to the design of this study. MFA organized the data collection and database, performed the statistical analyses, and wrote the first draft of the manuscript. MH wrote a major revision of the manuscript. All authors contributed to manuscript revision, read, and approved the submitted version.

## Conflict of Interest Statement

The authors declare that the research was conducted in the absence of any commercial or financial relationships that could be construed as a potential conflict of interest. The reviewer KL and handling editor declared their shared affiliation at the time of review.

## Appendix A

### Student Questionnaire

The proportion of students choosing each alternative is given in between brackets. For 5-point scales the mean is also presented. The correlations are between the students’ question response and the students’ frequency of written strategy choices on the pretest; statistically significant correlations are in bold.

1. How much do you like math? *not at all (0.10)/not so much (0.21)/it’s okay (0.45)/quite a bit (0.23)/a lot (0.01); M* = 2.85; *r*(144) = 0.06, *p* = 0.473.
2. How much effort do you put into math? *none (0.00)/not so much (0.01)/a bit (0.08)/quite a lot (0.53)/a lot (0.38); M* = 4.29; *r*(145) = 0.10, *p* = 0.236.
3. How good do you think you are at math? *not good at all (0.07)/not so good (0.25)/okay (0.46)/quite good (0.22)/very good (0.00); M* = 2.84; *r*(145) = 0.07, *p* = 0.385.
4. What is more important to you when you solve a mathematics problem? solving the problem quickly (0.02)/finding the correct solution (0.98); *r*(143) = 0.06, *p* = 0.487.
5. What is more important to you when you solve a mathematics problem? *being able to do it mentally (0.22)/being able to do it using paper (0.78);* ***r*(143) = 0.23, *p* = 0.005**.
6. How often do you solve problems without writing down a calculation? *almost never (0.16)/not often (0.29)/sometimes (0.40)/often (0.12)/very often (0.03); M* = 2.56; ***r*(145) = -0.19, *p* = 0.019**.
7. When you do not write down a calculation, why is that? *(tick boxes that apply)*.

      • because it is faster (0.41)
      • because then you get a correct solution more often (0.14)
      • because doing mental calculation shows you are smart (0.11)
      • because it is cooler to do mental calculation (0.01)
      • because you do not feel like writing anything down (0.22)
      • because you guessed the solution (0.14)
      • because it is not necessary to write down a calculation (0.49)

## Appendix B

### Teacher Questionnaire

The proportion teachers choosing each alternative is presented between brackets. For 5-point scales the mean is also presented. The correlations are between the question response and the frequency of the teachers’ students average percentage of written strategy choices on the pretest; statistically significant correlations are in bold.

1. Do you teach your students the whole-number-based algorithm, digit-based algorithm or non-algorithmic approaches for solving multidigit problems (such as 544÷34 or 12.6÷1.4)? When multiple approaches apply, tick multiple boxes. *whole-number-based algorithm (0.58)/both whole-number-based and digit-based algorithm (0.26)/digit-based algorithm (0.16); r*(17) = -0.18*, p* = 0.462.
2. To what extent do you as a teacher prefer a division algorithm? *strong preference whole-number-based - strong preference digit-based (5-point scale): M* = 3.0; *r*(17) = 0.06*, p* = 0.798.
3. To what extent do you as a teacher prefer an algorithmic over a non-algorithmic approach? *strong preference algorithmic - strong preference non-algorithmic (5-point scale): M* = 2.2; ***r*(17) = -0.46*, p* = 0.048.**
4. Which ability do you find more important in general for your students? *performing calculations well on paper - performing calculations well mentally (5-point scale): M* = 3.0; *r*(17) = -0.19*, p* = 0.444.
5. How often do you instruct your students in writing down intermediate steps or calculations? *almost never - daily (5-point-scale); M* = 4.2; *r*(17) = 0.297*, p* = 0.217.
6. What is more important to you when your students solve multidigit division problems? *(six 5-point scales)*.

      • *that they write down all calculations - that they try to do it mentally: M* = 2.4*; r*(17) = -0.26*, p* = 0.275.
      • *that they keep trying until they get the correct solution, even if that takes a lot of time - that they can do it quickly, even if they sometimes make mistake: M* = 2.5*; r*(17) = -0.29*, p* = 0.234.
      • *that they can determine the exact answer - that they can make a good estimation of the answer: M* = 3.5; *r*(17) = -0.10, *p* = 0.695.
      • *that they know one solution procedure - that they know multiple solution procedures: M* = 3.4; *r*(17) = 0.35, *p* = 0.14.
      • *that they use an algorithm - that they choose their own solution strategy: M* = 3.0; *r*(19) = -0.10, *p* = 0.687.
      • *that they use a method that can always be applied - that they use convenient shortcut strategies (such as* 1089÷11 = 1100÷11-1): *M* = 3.3; *r*(17) = -0.24, *p* = 0.320.
